# Logic circuits from zero forcing

**DOI:** 10.1007/s11047-014-9438-5

**Published:** 2014-07-26

**Authors:** Daniel Burgarth, Vittorio Giovannetti, Leslie Hogben, Simone Severini, Michael Young

**Affiliations:** 1 Department of Mathematics and Physics, Aberystwyth University, Aberystwyth, SY23 3BZ UK; 2 NEST, Scuola Normale Superiore and Istituto Nanoscienze-CNR, Piazza dei Cavalieri 7, 56126 Pisa, Italy; 3 Department of Mathematics, Iowa State University, Ames, IA 50011 USA; 4 American Institute of Mathematics, 360 Portage Ave, Palo Alto, CA 94306 USA; 5 Department of Computer Science, University College London, London, WC1E 6BT UK; 6 Department of Physics and Astronomy, University College London, London, WC1E 6BT UK

**Keywords:** Zero forcing, Logic circuits, Adiabatic quantum computation

## Abstract

We design logic circuits based on the notion of zero forcing on graphs; each gate of the circuits is a gadget in which zero forcing is performed. We show that such circuits can evaluate every monotone Boolean function. By using two vertices to encode each logical bit, we obtain universal computation. We also highlight a phenomenon of “back forcing” as a property of each function. Such a phenomenon occurs in a circuit when the input of gates which have been already used at a given time step is further modified by a computation actually performed at a later stage. Finally, we show that zero forcing can be also used to implement reversible computation. The model introduced here provides a potentially new tool in the analysis of Boolean functions, with particular attention to monotonicity. Moreover, in the light of applications of zero forcing in quantum mechanics, the link with Boolean functions may suggest a new directions in quantum control theory and in the study of engineered quantum spin systems. It is an open technical problem to verify whether there is a link between zero forcing and computation with contact circuits.

## Introduction

### Preliminaries

We order the two elements of a set $\Sigma =\{0,1\}$ such that $0<1$. This extends to a partial ordering on the set $\Sigma ^{n}=\{0,1\}^{n}$ by comparing words coordinate-wise. Let $x=x_{1},\ldots ,x_{n}$ and $y=y_{1},\ldots ,y_{n}$. Here, $x\succeq y$ means that $x_{i}\ge y_{i}$, for every $i=1,\ldots ,n$. A Boolean function $f:\Sigma ^{n}\longrightarrow \Sigma$ is *monotone* when $f\left( x\right) \ge\, f\left( y\right)$ if $x\succeq y$, for every $x,y\in \Sigma ^{n}$. Clearly, because of this, the symbol “$\succeq$” has a different meaning than majorization as a preorder of vectors.

Counted by the Dedekind numbers, monotone Boolean functions have an important role for proving lower bounds of circuit complexity (see, e.g., Leeuwen 1990, Chapter 14.4). Remarkably, any function obtained by composition of monotone Boolean functions is itself monotone. Examples of monotone Boolean functions are the conjunction AND and the disjunction OR. Indeed, every monotone Boolean function can be realized by AND and OR operations (but without NOT). Monotone Boolean functions are important in applications, for example, in the implementation of a class of non-linear digital filters called stack filters (Astola et al. 1997), in voting schemes, reliability theory, stability of hypergraphs, etc. Important methods for obtaining non-trivial bounds on specific monotone Boolean functions have been studied (see, e.g., the seminal work of Razborov 1985, and Alon and Boppana 1986).

The central topic of this paper is a connection between monotone Boolean function and zero forcing. The concept of *zero forcing* on graphs is a recent idea that is part of a program studying minimum ranks of matrices with specific combinatorial constraints (American Institute of Mathematics 2008). Zero forcing has been also called graph infection and graph propagation in the areas related to quantum dynamics and control theory of quantum mechanical systems (Burgarth and Giovannetti 2007; Severini 2008).

In quantum mechanics, zero forcing is a technique for determining the algebraic controllability of a many-body quantum system by operating on the particles of a proper subsystem only. The technique is important because it does not require the knowledge of the spectral properties of the physical operator governing the system, but only topological information about the interactions, i.e., the graph of the interactions.

Notice that, in the context described here, the term “zero forcing” seems to be unfortunate, because we are forcing ones, not zeros. However, we keep the term given that this is now the most commonly used in the literature. We will skip alternative definitions of zero forcing. The interested reader may see the references American Institute of Mathematics (2008), Burgarth et al. (2009), Burgarth and Giovannetti (2007).

For the purpose of formally describing zero forcing, we first need to define a *color-change rule*: if $G=(V,E)$ is a graph with each vertex colored either white or black, $u$ is a black vertex of $G$, and exactly one neighbor $v$ of $u$ is white, then change the color of $v$ to black. Given a coloring of $G$, the *final coloring* is the result of applying the color-change rule until no more changes are possible. Of course, the final coloring can include different vertices depending on the initial configuration. A *zero forcing set* for $G$ is a set $Z\subseteq V\left( G\right)$ such that if the elements of $Z$ are initially colored black and the elements of $V(G)\backslash Z$ are colored white, the final coloring of $G$ is all black.

In linear algebraic terms, zero forcing is related to certain minimum rank/maximum nullity problems of matrices associated to graphs (see American Institute of Mathematics 2008). As it usually happens for rank related questions, minimizing the size of zero forcing sets is a difficult combinatorial optimization problem, which turns out to be hard whose solution is hard even to approximate. (The PhD thesis of Aazami 2008, presents a detailed analysis.)

### Results

In the next section, we prove that zero forcing on graphs realizes all monotone Boolean functions, and highlight some simple related facts. The connection between zero forcing and circuits is obtained by associating a graph to each logic gate. We will show that the functions AND and OR are indeed easily realized by two different gadgets with a few vertices. This is not the first work observing that monotone Boolean functions can be realized in a combinatorial setting. For example, Demaine et al. (2012) have used the movements of a collections of simple interlocked polygons for the same purpose. Realizing general Boolean functions, or even some special classes, in non-standard computational models, has the potential of uncovering new mathematical ideas. These may help in practice for reformulating optimization problems, and more abstractly to establish lower bounds and to quantify resources. On the other side, we know that zero forcing can be directly used in the laboratory to optimize control operations on spin systems. For this reason, observing that the associated dynamical process is a computational primitive can be important to introduce parameters to quantify complexity of the physical evolution. With this aspects in mind, links between zero forcing and the abstract notion of computation are useful.

In Sect. 3, we describe the phenomenon of *back forcing* in the circuit. The phenomenon occurs when the color-change rule acts to modify the color of a vertex which has been already used during the computation. In some cases, back forcing implies that the information about the output of a Boolean circuit can be read not just by looking at the color of a *target* vertex corresponding to the final output of the process, but at the color of the vertices in certain intermediate or initial gadgets. The idea opens a simple but intriguing scenario consisting of many parties that perform computation in a distributed way: each party holds a subset of the gates and is able to read certain information about the input of other parties, since the color of its gates may have been modified by back forcing. Back forcing can be avoided by including some extra gadget acting as a filter. While we will not explore this idea in detail, we do believe that it is interesting and that it deserves further attention.

In Sect. 4, we show that zero forcing becomes *universal*, i.e., it can realize any Boolean function, if we apply a proper encoding. Specifically the *dual rail encoding*, where two vertices are assigned to each logical bit, is a method to construct the NOT gate and therefore to obtain universal computation. In this way, we can implement reversible computation. It is interesting to remark that while the dynamics of zero forcing is irreversible, the “higher level” process can also be used in a reversible manner. Conclusions are in Sect. 5.

## Main result

For the sake of completeness, let us recall the behaviour of the logic gates/functions AND and OR: AND outputs $1$ if and only if the input is $(1,1)$; OR outputs $0$ if and only if the input is $(0,0)$. In our model, logical bits correspond to specific vertices of a graph. Conventionally, the logical value $0$ is a white vertex; $1$ is a black vertex. Once provided the relevant definitions in the previous section, our main result is easy to prove:

### **Theorem 1**

*Zero forcing realizes all monotone Boolean functions*.

### *Proof*

It is sufficient to show that zero forcing realizes the functions AND and OR.

**Fig. 1:**
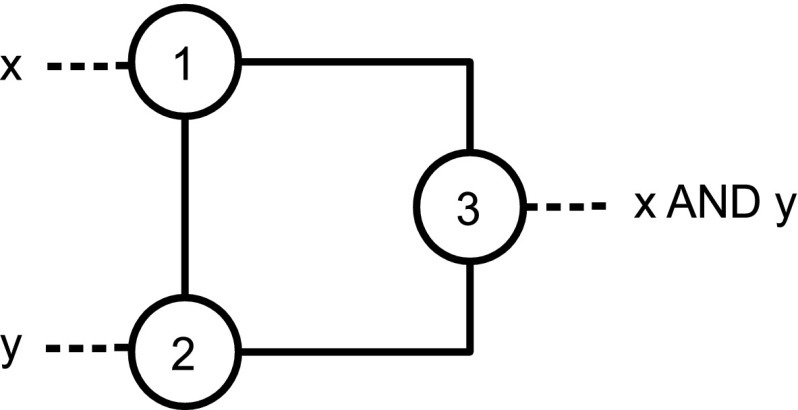
The gate for the function AND

**Claim 1** *The gate AND is realized by the gadget* $G_{\text {{AND}}}$ *with vertices* $\{1,2,3\}$ *and edges* $\{\{1,2\},\{1,3\},\{2,3\}\}$,* where* $1$ *and* $2$ *are the input vertices and* $3$ *is the output vertex, containing the result and being able to propagate the color. All vertices are initially colored white. An illustration of the gadget* $G_{\text {{AND}}}$ *is in Fig.* 1.

*Proof of Claim 1* If no action is taken then the final coloring of the gadget is white. If we color vertex $1$ black then the final coloring is all white but for vertex $1$. The same holds for vertex $2$. However, if we color vertex $1$ and vertex $2$ black then the color-change rule implies that vertex $3$ is black at step $2$. In fact, $\{1,2\}$ is a zero forcing set for $G_{\text {{AND}}}$.

**Fig. 2:**
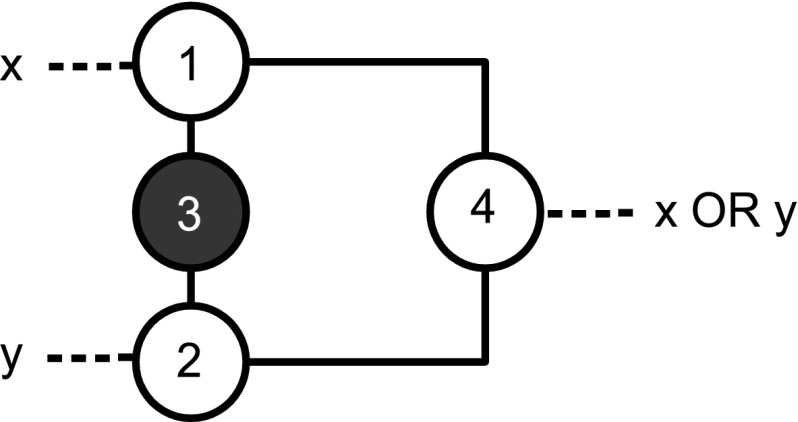
The gate for the function OR

**Claim 2** *The gate OR is realized by the gadget* $G_{\text {{OR}}}$ *with vertices* $\{1,2,3,4\}$ *and edges* $\{\{1,3\},\{1,4\},\{2,3\},\{2,4\}\}$,* where* $1$ *and* $2$ *are the input vertices. The output vertex is vertex* $4$.* Vertex* $3$ *is initially colored black. See Fig.* 2.

*Proof of Claim 2* If no action is taken then the final coloring of the gadget is all white, but for vertex $3$. If we color vertex $1$ black then the color-change rule implies that vertex $4$ is black at step $2$. The same holds for vertex $2$ and for vertex $1$ and vertex $2$ together. In fact, $\{1,3\},\{2,3\},\{1,2,3\}$ are zero forcing sets for $G_{\text {{OR}}}$, able to propagate the color for inducing the next step of the computation.

It is important to observe that zero forcing does not realize the function NOT, since when a vertex is colored black, it can not change color anymore. The consequence is that zero forcing does not realize universal computation (any Boolean function can be implemented using AND, OR and NOT gates) but monotone Boolean functions only. This concludes the proof. $\square$

**Fig. 3:**
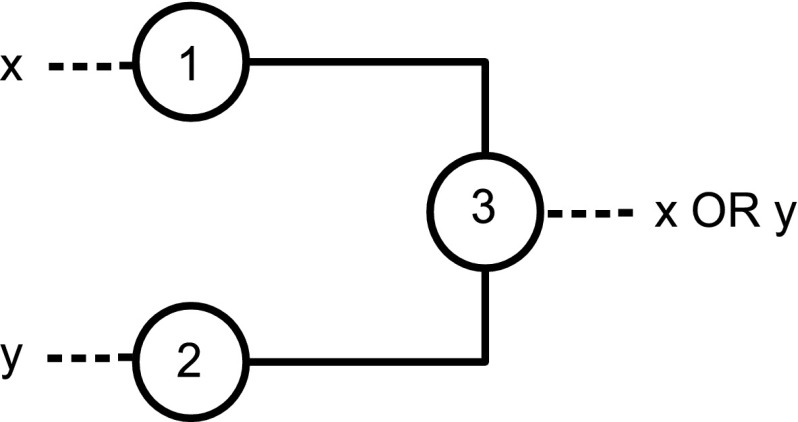
A gate for the function OR, where color-change rule does not move the input forward

**Fig. 4:**
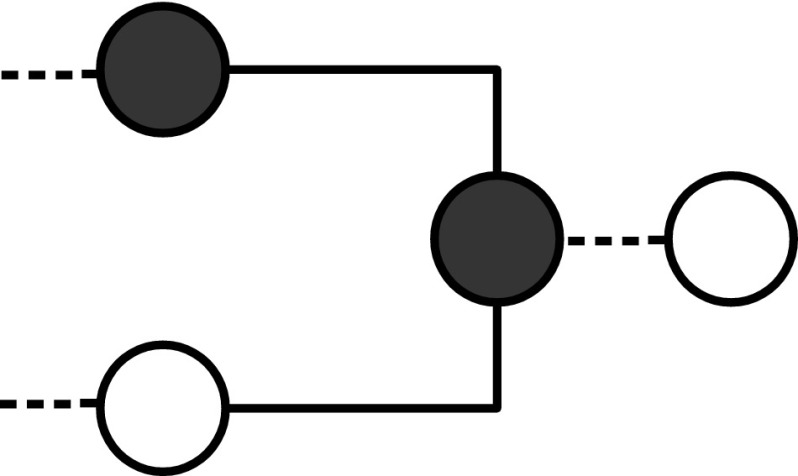
The figure shows that an OR gate in which all vertices are initially white does not move the input forward

**Fig. 5:**
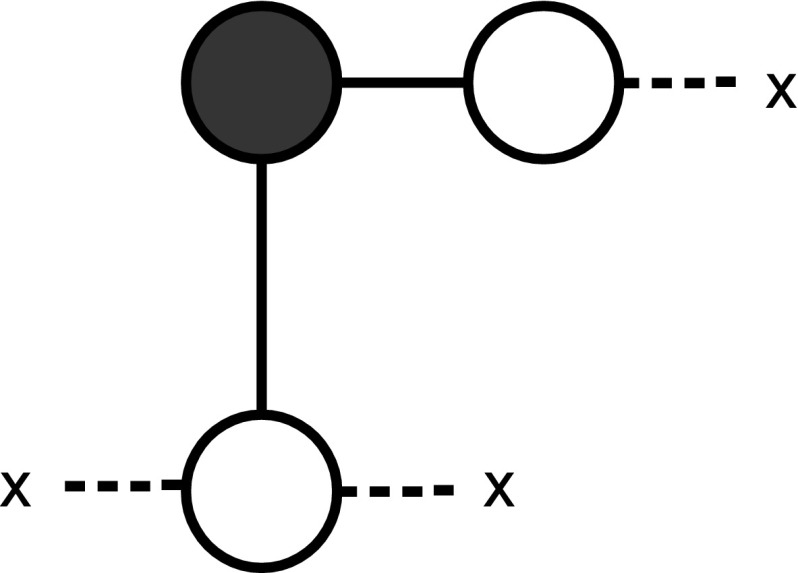
The gate for the function COPY

It may be worth observing the following points:

- Notice that extra vertices forming *delay lines* may be needed to assemble a circuit such that the output produced by zero forcing in parallel gates is synchronous. However, given our choice of gadgets, exactly $2$ time steps are required for output of zero forcing in $G_{\text {{AND}} }$ and $G_{\text {{OR}}}$. At time step $3$ the color-change rule acts on the next gate in the circuit. There is then a convenient distinction between internal and external time: *internal time* refers to the zero forcing steps inside the gadgets/gates; *external time* refers to the time steps of the computation.
- The gadgets $G_{\text {{AND}}}$ and $G_{\text {{OR}}}$ have three and four vertices, respectively. By inspection on all possible combinations of white and black vertices for graphs with at most four vertices, we can observe that we have chosen the smallest possible gadgets, in terms of number of vertices and edges, realizing the two functions. One might think that the gate OR is realized also by the gadget with three vertices in Fig. 3. Although the gadget implements the OR correctly, it cannot be used as an initial or intermediate gate of a circuit, since in this gadget the color-change rule does not move forwards the output to the next gate, but it halts at vertex $3$. See Fig. 4.
- Let us consider the gadget $G_{\text {{OR}}}$. If we color vertex $1$ black then the color-change rule implies that vertex $4$ is black at step $2$. Suppose that vertex $2$ is colored white at step $1$. At step $3$ the gate has computed the OR function in vertex $4$ with input $\{0,1\}$. At step $3$ vertex $2$ is also colored black under the action of the color-change rule, because this is the unique white neighbour of vertex $3$. This is necessary in order for the computation to proceed using the output (black vertex $4$). So, for all inputs with output $1$, the vertices of $G_{\text {{OR}}}$ are black after two steps of the internal time. Such behaviour is discussed in more detail in the next section.
- It is straightforward to realize the operation COPY. See Fig. 5.

## Back forcing

**Fig. 6:**
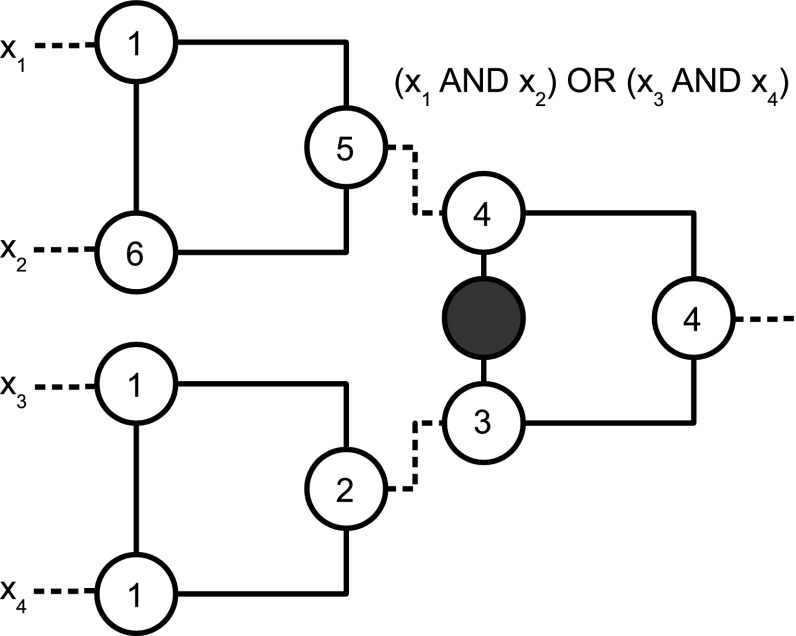
A circuit computing the Boolean function $$(x_{1}$$ AND $$x_{2})$$ OR $$(x_{3}$$ AND $$x_{4})$$. The circuit exhibits the phenomenon of back forcing

If each Boolean variable in the input of a circuit is set to $1$, then the vertices of the circuit that are initially colored black form a zero forcing set. However, this is not the only situation in which we have a zero forcing set. Figure 6 gives an example.

This is a circuit computing the Boolean function $$(x_{1}$$ AND $$x_{2})$$ OR $$(x_{3}$$ AND $$x_{4})$$. The number in the vertices of the figure specify the internal time step at which the vertex is black; the vertices labeled by $$1$$ are initially colored black. The output of the circuit is $$1$$ at step $$4$$ and at step $$6$$ of the internal time the vertices encoding the input of the function are all colored black. This can happen if and only if three of the input vertices are colored white at internal time $$1$$.

**Fig. 7:**
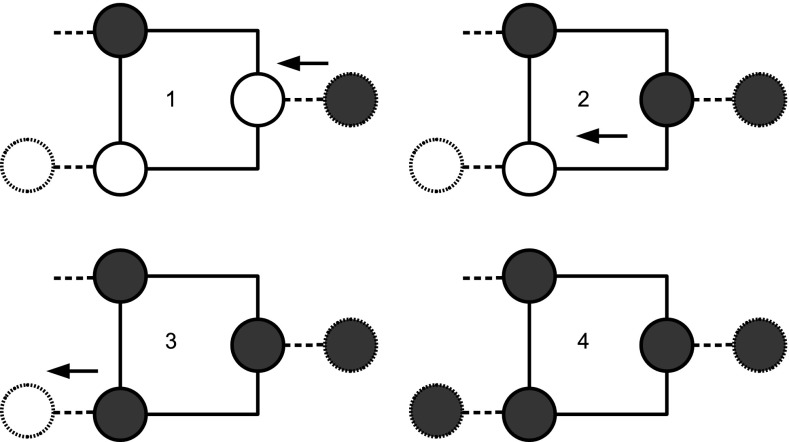
The steps of back forcing

The phenomenon will be called *back forcing*, because it is induced by the color-change rule acting backwards with respect to the direction from input to output in the whole circuit. The gadget $G_{\text {{AND}} }$ exhibits back forcing conditionally on having input $\{0,1\}$. The type of back forcing in $G_{\text {{AND}}}$ can be called *transmittal back forcing*, because if something back forces its output black then the gate transmits the back force, i.e., it modifies the color of the output vertex in a gate used previously. Figure 7 clarifies the dynamics.

The gadget $$G_{\text {{OR}}}$$ needs to force an input forward in order to color black one of the output vertices adjacent to its inputs and in another gate. In this sense, $$G_{\text {{OR}}}$$ does not have transmittal back forcing. In other words, a gate at external time $$t$$, can not back force its color into $$G_{\text {{OR}}}$$ at external time $$t+1$$. In contrast, the circuit $$(x_{1}$$ AND $$x_{2})$$ OR $$(x_{3}$$ AND $$x_{4})$$ can initiate back forcing as described above (when it an intermediate element in the circuit).

We can also slow down back forcing, by including appropriate *delay lines*—for example, by adding extra vertices in each gadget or between them. Alternatively, we could consider delay lines directly embedded in the structure of the gadgets implementing the logical gates.

**Fig. 8:**
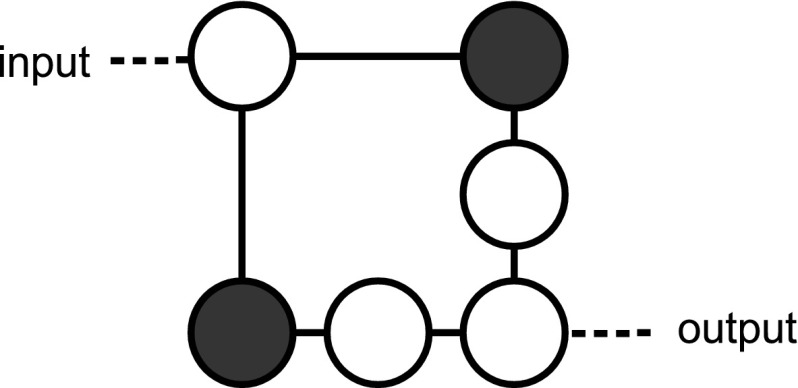
A gadget acting as a filter: its role is to avoid back forcing

Also, back forcing can be avoided completely by including the gadget in Fig. 8. The gadget acts as a *filter*. In some sense, the filter can be understood as an *electronic diode* allowing zero forcing only in one direction.

In relation to the circuit for the function $$(x_{1}$$ AND $$x_{2})$$ OR $$(x_{3}$$ AND $$x_{4})$$, it may be interesting to see that if there are two parties each one choosing the input of one of the two AND gates, and each one having access to only the corresponding vertices, given the back forcing, the parties can then learn the output of the circuit by looking at the color of their vertices at the end of the computation, except when a party chooses $$(0,0)$$ (i.e., white, white).

## Universality

Despite the fact that the color-change rule induces a non-reversible process (black coloring cannot be undone) a simple modification of the encoding strategy allows us to implement universal, and hence also reversible, computation (see Drechsler and Wille 2011; Saeedi and Markov 2013; Vitanyi 2005 for detailed reviews on this topic).

**Fig. 9:**
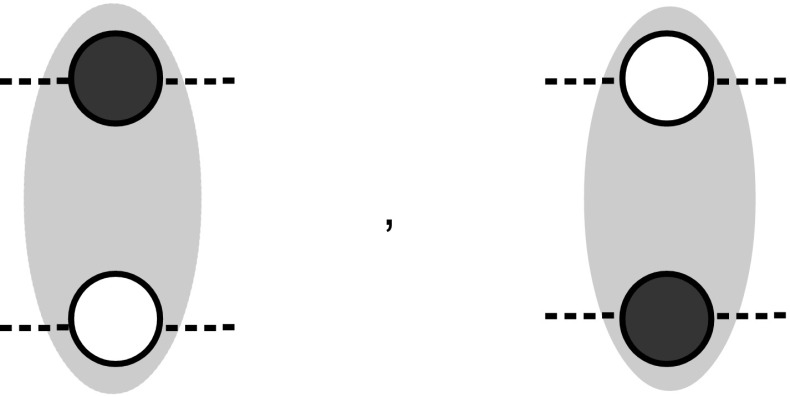
Physical bits for $0$ and $1$ in a dual rail encoding

**Fig. 10:**
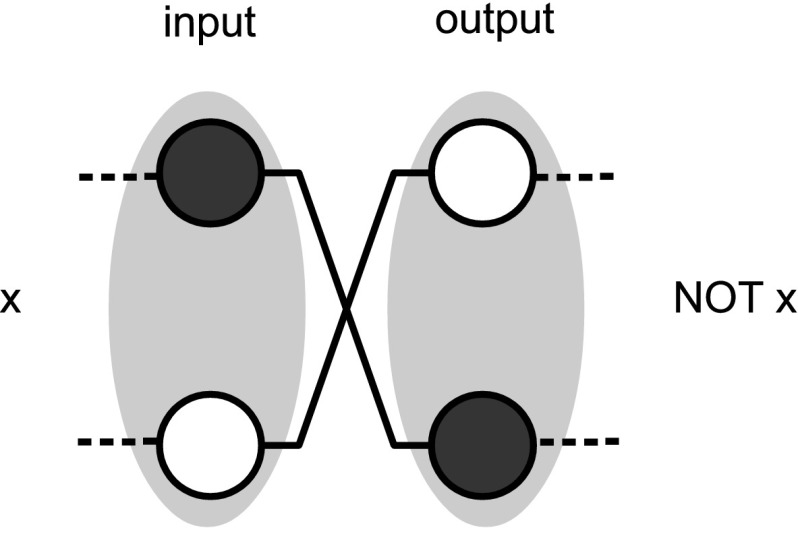
In a dual rail encoding the logical NOT can be implemented by swapping the physical bits

The idea is to adopt a *dual rail* strategy, where two vertices are employed to encode a single *logical bit*. Specifically, as shown in Fig. 9, in this scheme we associate the logical bit 0 to a configuration in which (say) the first vertex is colored in black while the second is kept white, and the logical 1 to the opposite configuration (i.e. the first vertex being left white and the second one being colored black). With such encoding we can now design the gate NOT by simply drawing a graph in which the nodes are exchanged at the output (see Fig. 10). Also a dual rail AND gate can be easily realized. Universal computation is hence achieved by constructing a NAND gate via concatenation of AND with NOT and by observing that the COPY gate for the dual rail encoding is simply obtained by just applying to both the nodes that form a bit the transformation of Fig. 5. Once universal computation has been achieved, we can easily turn it into a reversible one, e.g., by building a Toffoli gate (Toffoli 1980). This to remark that even if zero forcing is an irreversible process, it can still be used to induce a reversible computational dynamics.

## Conclusions

We have shown that all monotone Boolean functions can be realized by zero forcing in a graph constructed by *gluing* together the copies of two types of subgraphs/gadgets corresponding to the Boolean gates AND and OR. We have briefly discussed the minimality of such gadgets in terms of vertices and edges. Even we did not give a formal proof, it seems evident that our gadgets are optimal in this respect.

We have highlighted a phenomenon of “back forcing action”. Back forcing has an effect on the coloring of gates already used, as a function of what has happened in the “future”, i.e., at a later stage of the computation. Because of the relation between zero forcing and minimum ranks, the model described here is amenable to be studied with linear algebraic tools, potentially suggesting a novel direction in the analysis of monotone Boolean functions and, speculating, the introduction of rank problems in questions relevant to parametrized complexity.

Finally, we have shown that universal computation can be obtained with zero forcing by simply adopting a dual rail encoding.

An open direction suggested by the paper is to understand the link between zero forcing and the dynamics at the basis of other unconventional models of computation, like, for example, the billiard ball computer—introduced as a model of reversible computing (Fredkin and Toffoli 1982)—, models involving geometric objects, and dominos (Demaine et al. 2012). A more precise open technical problem consists of verifying whether there is a link between zero forcing and computation with contact circuits (Red’kin 1979).

From a physics perspective, zero forcing sets describe controlling, cooling, and symmetries of quantum systems—engineered spin systems like Heisenberg and AKLT chains. While an application of monotone Boolean functions in physics is not immediate, the connection that we have highlighted is nonetheless interesting. For example, can monotone Boolean functions be used to characterize symmetries in quantum mechanics?
